# Notch mediates the glycolytic switch via PI3K/Akt signaling to support embryonic development

**DOI:** 10.1186/s11658-023-00459-4

**Published:** 2023-06-26

**Authors:** Heng Wang, Wenqi Liang, Xuyang Wang, Yuchun Zhan, Wence Wang, Lin Yang, Yongwen Zhu

**Affiliations:** Guangdong Provincial Key Laboratory of Animal Nutrition and Regulation, College of Animal Science, South China Agricultural University, Guangzhou, 510000 China

**Keywords:** Notch signaling, Glycolysis, PI3K/Akt, Avian embryonic development

## Abstract

**Background:**

Energy metabolism disorder or insufficient energy supply during incubation will affect the development and survival of avian embryos. Especially, β-oxidation could not provide the continuous necessary energy for avian embryonic development due to the increasing energy demand under hypoxic conditions during the mid–late embryonic stages. The role and mechanism of hypoxic glycolysis replacing β-oxidation as the main source of energy supply for avian embryonic development in the mid–late stages is unclear.

**Results:**

Here, we found that in ovo injection with glycolysis inhibitor or γ-secretase inhibitor both decreased the hepatic glycolysis level and impaired goose embryonic development. Intriguingly, the blockade of Notch signaling is also accompanied by the inhibition of PI3K/Akt signaling in the embryonic primary hepatocytes and embryonic liver. Notably, the decreased glycolysis and impaired embryonic growth induced by the blockade of Notch signaling were restored by activation of PI3K/Akt signaling.

**Conclusions:**

Notch signaling regulates a key glycolytic switch in a PI3K/Akt-dependent manner to supply energy for avian embryonic growth. Our study is the first to demonstrate the role of Notch signaling-induced glycolytic switching in embryonic development, and presents new insight into the energy supply patterns in embryogenesis under hypoxic conditions. In addition, it may also provide a natural hypoxia model for developmental biology studies such as immunology, genetics, virology, cancer, etc.

**Graphical Abstract:**

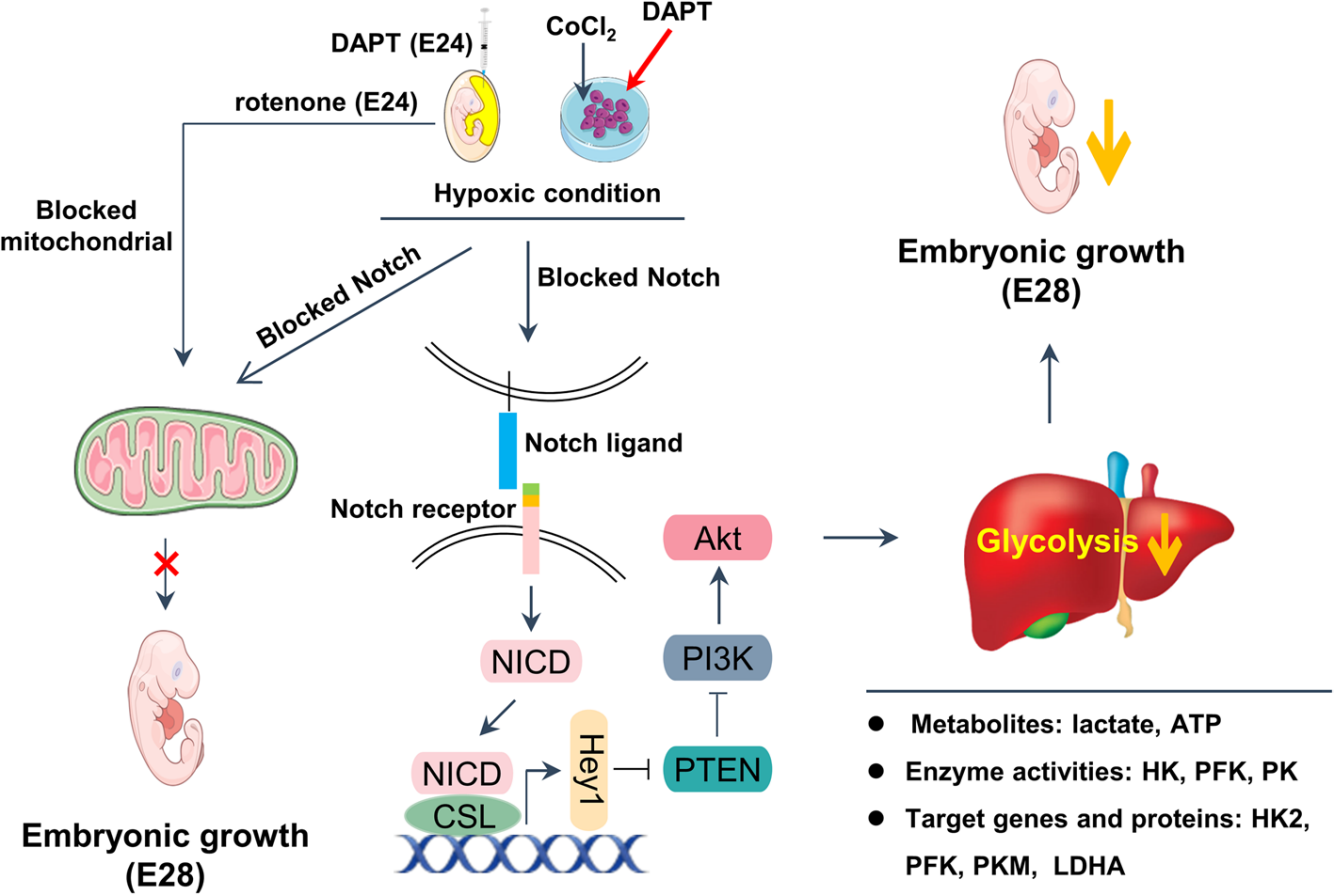

**Supplementary Information:**

The online version contains supplementary material available at 10.1186/s11658-023-00459-4.

## Highlights

- Glycolysis plays a dominant role in the energy supply to support mid–late avian embryonic growth.
- Notch signaling regulates a switch to glycolysis in the liver.
- Notch switched glycolysis in a PI3K/Akt-dependent manner in vivo and in vitro.

**Supplementary Information:**

The online version contains supplementary material available at 10.1186/s11658-023-00459-4.

## Introduction

The low hatchability of breeding eggs is an important factor limiting the development of the meat goose industry in China [1]. Unlike the mammalian embryo, the avian embryo develops in an egg that forms a naturally closed chamber [2]. Energy metabolism disorder or insufficient energy supply will affect the development and survival of geese embryos [3]. Dramatic changes in metabolic patterns occur during avian embryogenesis to satisfy different physiological demands of growth [4]. Energy metabolism is vital for maintaining embryonic development, and as the mid–late period coincides with rapid organic development, enhanced oxygen consumption, and increased glycogen storage for pipping and emergence, the energy is heavily utilized [3, 5, 6]. For example, as the embryo develops and O_2_ diffusion across the air–cell membrane increases during the early–mid incubation period [7], the substrates that the embryo metabolizes for energy shift from hypoxic glycolysis of carbohydrates (respiratory quotient, RQ ∼1.0) to β-oxidation of fatty acids (RQ ∼0.7) [8]. In the transition from mid to late incubation, β-oxidation cannot provide all the necessary energy due to the increasing energy demand under hypoxic conditions [7, 9]. We speculate that hypoxic glycolysis might replace β-oxidation and act as the dominant mode of energy supply for avian embryos during the mid–late developmental stages. Thus, studies of hypoxic glycolysis in avian embryogenesis are important for economic developments in poultry breeding, which uses agriculturally significant avian species to provide humans with high-quality animal proteins (e.g., meat and eggs). Compared with other domestic fowl embryos, geese embryos grow fast, have sensitive responses to changes in energy supply, and have longer incubation periods (21 days for chickens versus 28 days for ducks and 30 days for geese). Thus, geese embryos serve as an ideal model to study the role of hypoxic glycolysis in avian embryonic development.

The Notch signaling pathway is a conserved signaling pathway that mediates juxtacrine cell–cell communication [10] and is involved in many key cellular decisions and other core processes during embryogenesis [11]. For example, Notch signaling is required for the generation of hematopoietic stem cells in mice embryos [12] and the induction of central nervous system development in embryos of *Drosophila melanogaster* [13]. Although two Notch proteins of Notch1 and Notch2 of geese are annotated in the National Center for Biotechnology Information (NCBI), the role and underlying mechanisms of Notch signaling in chicken embryogenesis, particularly in the switch to hypoxic glycolysis to provide energy for avian embryonic development are poorly understood. In the present study, the role of glycolysis in embryonic development and the potential mechanisms of Notch signaling in regulating hypoxic glycolysis were evaluated in avian embryos. The results are the first to demonstrate that Notch signaling, in a PI3K/Akt-dependent manner, induces a switch to hypoxic glycolysis, which serves as the main pathway of energy supply during the mid and late stages of avian embryonic development.

## Materials and methods

The animal care and use protocol was approved by the Institutional Animal Care and Use Committee of South China Agricultural University (SCAU-10564), and the study was performed following the Regulations for the Administration of Affairs Concerning Experimental Animals.

### Incubation procedure

Fertilized geese eggs with similar egg weights were obtained from the same age of breeding female geese fed the same diet to ensure similar nutrient deposition in the yolk for embryonic development. Eggs were incubated in a microcomputer automatic incubator (Keyu, Shandong, China) at a relative humidity of 65 ± 5% and a temperature of 37.5 ± 0.5 °C. During incubation, eggs were turned at an angle of 60° every 1.5 h until embryonic day (E)28, and then all eggs were transferred to the hatcher with no further egg turning until hatching on E30. Notably, cracked eggs and dead embryos were removed following candling on E8 and E18, and all hatched eggs were wetted and cooled with 38 °C water twice per day from E9 to E28 to eliminate the adverse effects of increased heat production on embryonic development, as described previously [14]. Other incubation management protocols were carried out according to normal incubation procedures. Ten egg embryos, representing the weight distribution of the eggs at the set, were selected on E16, E19, E22, E25, and E28. Embryos were killed by cervical dislocation, and then the egg embryos, yolk sac, and liver were weighed to calculate the average values per replicate from each incubation time. Liver samples were collected for the measurement of indices related to energy metabolism to study the developmental changes in glycolysis and Notch signaling during the mid–late incubation stages.

### In ovo injection experiment

The in ovo injection procedure was carried out as described previously [15]. Before injection, the border of the air sac or the yolk sac was identified under candlelight. The fertilized eggs were disinfected with 75% ethanol in the insertion region before injection. A sterile disposable 25.0 × 0.6 mm needle was attached to a 1.0 mL syringe, which was replaced after each egg injection. The holes were sealed with medical adhesive tape (1.0 × 1.0 cm^2^) immediately after injection. Eggs from the noninjection group were placed outside the incubator environment for 5 min for standardization. Finally, the eggs from each replicate of each treatment were placed on the same egg tray and incubated at 37.5 ± 0.5 °C with a relative humidity of 65 ± 5%. The in ovo injection experiment was carried out as mentioned above at the developmental window of glycolysis on E24. The effects of 2-deoxy-d-glucose (2-DG, glycolysis inhibitor), rotenone (mitochondrial electron transport chain complex I inhibitor), *N*-[*N*-(3,5-difluorophenacetyl)-l-alanyl]-*S*-phenyl glycine t-butyl ester (DAPT, γ-secretase inhibitor), LY294002 (an inhibitor available for the PI3K family), and 740 Y-P (PI3K family agonist) were tested by in ovo injection to induce avian embryonic models.

### Isolation, culture, identification, and hypoxia induction of primary hepatocytes

Hepatocytes were isolated from geese embryos on E24 according to the procedure reported by Osman et al. [16]. In brief, liver tissue was digested with 3 to 5 volumes of 0.1% type IV collagenase (C4-28, Merck, USA) at 37 °C for 20 min. Next, the digestion process was terminated by adding an equal volume (v) of complete medium containing high-glucose Dulbecco’s modified Eagle medium (DMEM, Sigma, D0822), 100 IU/mL penicillin, 100 µg/mL streptomycin (Gibco 15140122), 2 mM glutamine (Sigma, G7513), 10% fetal bovine serum (Gibco, 10099141), and 0.02 mL/L EGF (Sigma, E9644) to the mixture. After subsequent centrifugation at 700×*g* for 8 min, the cells were suspended in a complete medium and seeded in cell culture flasks. Hepatocytes were identified by immunofluorescence as described previously [17, 18]. Cell morphology was observed under an inverted microscope (DMIL LED, Leica, Germany). A CCK-8 kit (CA1210, Solarbio Co., Ltd., Beijing, China) was used to determine the viability of hepatocytes. Cell proliferation was measured by a 5-ethynyl-2′-deoxyuridine (EdU) assay using a BeyoClick™ EdU-488 Cell Proliferation Kit (C0071S, Beyotime Biotechnology Co., Ltd., Shanghai, China). According to the description in a previous study, CoCl_2_ was selected as a hypoxia mimetic agent to establish the hypoxic environment and metabolic adaptation of primary hepatocytes in vitro [19]. Primary hepatocytes were seeded in six-well plates for 24 h and were then exposed to 0, 100, 200, 300, 400, or 500 µM CoCl_2_ for 24 h in a cell incubator with 5% CO_2_ at 37 °C to determine the optimum concentration for simulating the hypoxic environment. Each experiment was repeated three times.

### Targeted metabolomic analysis

Targeted energy metabolomic profiling of the liver was performed by Applied Protein Technology Co., Ltd. (Shanghai, China) using a liquid chromatography tandem mass spectrometry (LC‒MS/MS) system. In brief, 60 mg of the sample was homogenized with 500 µL of a precooled methanol/acetonitrile/H_2_O (2:2:1, v/v/v) solution and vortexed for 60 s. The mixture was then sonicated on ice for 30 min and left at −20 °C for 1 h to precipitate proteins. Next, the mixture was centrifuged at 14,000×*g* for 15 min at 4 °C, and the supernatant was dried using a lyophilizer. The dried protein extracts were then dissolved in 100 µL of acetonitrile/H_2_O (1:1, v/v) solution and centrifuged at 14,000×*g* for 15 min at 4 °C. The analysis was performed with Ultra High-Performance Liquid Chromatography Systems (1290 Infinity LC, Agilent Technologies) and a QTRAP Mass Spectrometer (AB SCIEX 5500). In brief, mobile phases A and B were 10 mM ammonium acetate solution and acetonitrile, respectively. The sample was placed in an autosampler at 4 °C with the column temperature at 45 °C, a flow rate of 0.3 mL/min, and an injection volume of 2 µL. Gradient of mobile phase B: 90–40% B at 0–18 min, 40–90% B at 18–18.1 min, and 90–90% B at 18.1–23 min. In addition, the MS system operated in negative ion mode at the following conditions: ion sapary voltage floating, −4500 V; source temperature, 450 °C; ion source gas 1, 45; ion source gas 2, 45; curtain gas, 30. Analyses were determined by electrospray ionization using multiple reaction monitoring. Peak chromatographic area and retention time were analyzed with Multiquant software.

### Biochemical indices related to glycolysis

Glucose and lactate levels were determined with a glucose kit (A154-1-1) and a lactate kit (A019-2-1) (Nanjing Jiancheng Bioengineering Institute, Nanjing, China), respectively. The ATP levels in the liver and hepatocytes were determined using an ATP detection kit according to the manufacturer’s instructions (S0026, Beyotime Biotechnology Co., Ltd., Shanghai, China). The activities of the rate-limiting enzymes hexokinase (HK) (A077-3-1), phosphofructokinase (PFK) (A129-1-1), and pyruvate kinase (PK) (A076-1-1) were determined by kits (Nanjing Jiancheng Bioengineering Institute, Nanjing, China) according to the manufacturer’s instructions. The oxygen consumption rate was measured using an Oxygen Consumption Rate Assay Kit (600800, Cayman Chemical, Ann Arbor, MI, USA).

### Quantitative RT–PCR

Total RNA was isolated from liquid nitrogen-frozen liver or hepatocytes using the RNA Purification Kit (B0004DP, EZBioscience Co., Ltd., Beijing, China). The quantity and purity of RNA were assessed using a NanoDrop 1000 spectrophotometer (Thermo Fisher Scientific, Waltham, MA, USA), and agarose gel electrophoresis was used to test the quality of RNA. Total RNA was reverse transcribed into cDNA by the Color Reverse Transcription Kit (A0010CGQ, EZBioscience Co., Ltd., Beijing, China), and a 2× Color SYBR Green qPCR Master Mix kit (A0012-R2, EZBioscience Co., Ltd., Beijing, China) was used for subsequent RT‒PCR amplification on a Bio-Rad C1000 Touch Thermal Cycler (Bio-Rad, Pleasanton CA, USA). The primers were designed and synthesized by Sangon Biotech Co., Ltd. (Shanghai, China), and the sequences were as follows:

- *HIF1α*: 5′ATGCCGAAGAAGCAAGGAGT3′ and 5′TCCATTTTGGCTTCTGTCTCC3′;
- *Notch1*: 5′ATCTCATCAACTGCCACGCA3′ and 5′AGCGGGGTCTCCTCCTTATT3′;
- *Notch2*: 5′CCAGGGTTCACGGGAGA3′ and 5′TTGGCACAAGGTTGAGATG3′;
- *Dll1*: 5′CTTTTGCGACAAACCTGGGG3′ and 5′CTGGTTGCAGAAAAGGCCAC3′;
- *Dll4*: 5′CTGGTGGCTTTGCTCATA3′ and 5′GATTCTTGGGTTTGTAGTTTG3′;
- *Jag1*: 5′AAACACCCAAACTGGAC3′ and 5′GCTCTTGAGTGCCTTTAT3′;
- *Jag2*: 5′TGGAAGGTTGGATGGGAGAA3′ and 5′ACAGCCTGGGTAGCGGACAC3′;
- *Hey1*: 5′GCGGGAGGGAAAGGTTAT3′ and 5′AGGCGTAGTTATTGAGATGAGA3′;
- *HK2*: 5′GGCAGTCGCTTTCTATCTGG3′ and 5′GAAGATGATCAGCGGGATGT3′;
- *PKF*: 5′TCTACAACCTCTACTCCTCC3′ and 5′CTTCTTCCTCAGTCCGATCA3′;
- *PKM*: 5′CTACAGACCTGTGGCTATTG3′ and 5′GAGATTCTTGTAGTCCAGCC3′;
- *NDUFA5: *5′CACTGGGCTCGTAGGATTGG3′ and 5′TGCACCAAATTAAGCCGCTG3′;
- *LOC106045434: *5′GGACTGGAGTAACTCAATGG3′ and 5′GCTATACTTCAGAGGTCCTG3′;
- *LOC106044242: *5′CAACAAGCCAGATATCGACG3′ and 5′GGCCTAAGTTCCTGGATAAC3′;
- *Glut1*: 5′CCAAGAGTGTCCTCAAGAAG3′ and 5′GGTGGAGTAGTAGAAAACCG3′;
- *GAPDH*: 5′TCTGTCGTGGACCTGACCTGC3′ and 5′GCCAGCACCCGCATCAAA3′;
- *LDHA*: 5′CCTTTCTGTGGCAGATCTAG3′ and 5′GTAGTTCCTTCTGGATTCCC3′;
- *PTEN*: 5′GAAGACCATAACCCACCAC3′ and 5′GGTCCTTACTTCCCCATAGA3′;
- *PI3K*: 5′ACCCAAGCGAGGATGAGG3′ and 5′TGTTGCCCGTGTTGAATG3′;
- *Akt*:5′TGCTGGATAAAGATGGAC3′ and 5′CTGGTTGTAGAAAGGGAG3′;
- *β-actin:* 5′CCATTGGCAATGAGAGGTTC3′ and 5′TGGATACCGCAGGACTCCATA3′;

### Western blot analysis

Total protein from the liver or hepatocytes was extracted with radioimmunoprecipitation assay (RIPA) lysis buffer containing 1 mM phenylmethanesulfonyl fluoride (ST506, Beyotime Biotechnology, Shanghai, China). The protein concentration was measured with a bicinchoninic acid (BCA) kit (A045-4-2, Nanjing Jiancheng Bioengineering Institute, Nanjing, China). The lysates were heated at 100 °C for 10 min and then stored at −20 °C. The proteins in the lysates were separated by electrophoresis on 10% sodium dodecyl sulfate–polyacrylamide gel electrophoresis (SDS‒PAGE) and were then transferred onto polyvinylidene difluoride (PVDF) membranes. Next, the membranes were blocked in 5% skim milk for 2 h before being washed three times with tris-buffered saline and polysorbate 20 (TBST) for 10 min each. The primary antibodies used in the present study were mouse anti-β-actin (66009-1-Ig, Proteintech, Wuhan, China), rabbit anti-HIF-1α (WL01607, Wanleibio, Shenyang, China), rabbit anti-Hey1 (19929-1-AP, Proteintech, Wuhan, China), rabbit anti-NICD (WL03097a, Wanleibio, Shenyang, China), mouse anti-GAPDH (HC301-01, TransGen Biotech, Beijing, China), rabbit anti-Glut1 (WL03141, Wanleibio, Shenyang, China), rabbit anti-HK2 (WL02454, Wanleibio, Shenyang, China), rabbit anti-PFK (P17858, Cusabio, Wuhan, China), rabbit anti-PKM (D120008, Sangon Biotech, Shanghai, China), rabbit anti-LDHA (D164055, Sangon Biotech, Shanghai, China), rabbit anti-PFKFB2 (D161387, Sangon Biotech, Shanghai, China), rabbit anti-PI3K (WL03380, Wanleibio, Shenyang, China), rabbit anti-Akt (WL0003b, Wanleibio, Shenyang, China), and p-Akt (4060S, Cell Signaling Technology, Massachusetts, USA). The secondary antibodies used in the present study were Horseradish Peroxidase (HRP) goat anti-mouse IgG (SA00001-1, Proteintech, Wuhan, China) and HRP goat anti-rabbit IgG (SA00001-2, Proteintech, Wuhan, China). The PVDF membranes were incubated in enhanced chemiluminescence (ECL) substrate for 2 min and were then visualized by chemiluminescence (5200, Bio-Tanon, Shanghai, China).

### Statistical analysis

All data were analyzed using GraphPad Prism version 8.3.0 (GraphPad Software, USA), and a one-way ANOVA followed by Tukey’s multiple comparison test was used to compare differences between groups. All data are presented as the mean ± SEM. The level of significance was set at *P* < 0.05, *P < 0.05, **P < 0.01;, ***P < 0.001, and ****P < 0.0001.

## Results

### Glycolysis is the main mode of energy supply during the mid–late embryonic stages

**Fig. 1:**
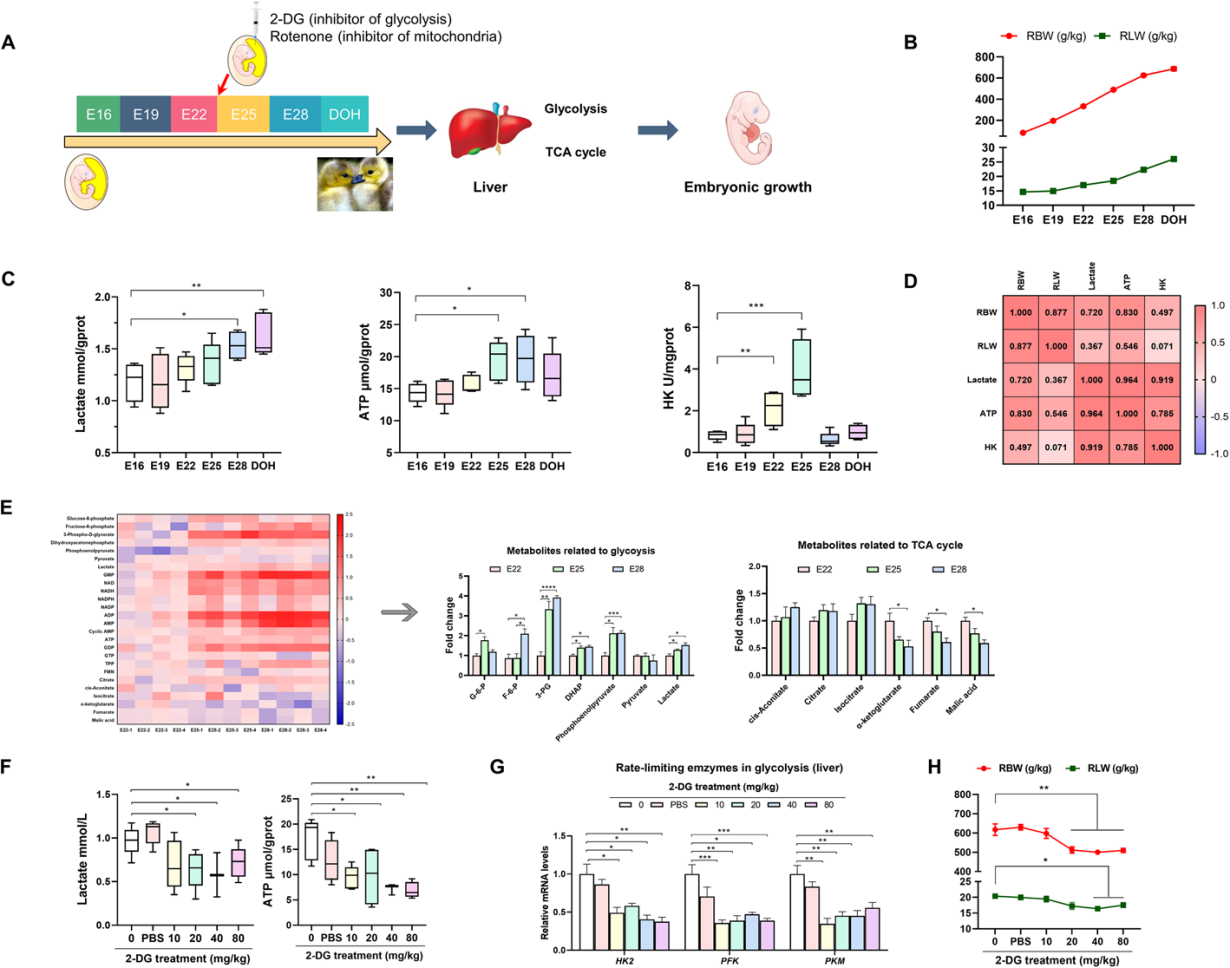
Glycolysis acted as the main energy supply mode during the mid–late embryonic stages. **A** Schematic depiction of cross-talk between glycolysis and avian embryonic growth. **B** Developmental changes of the geese embryos from E16 to DOH (*n* = 10). **C** Developmental changes in hepatic lactate, ATP content, and activity of HK from E16 to DOH (*n* = 6). **D** Correlation between hepatic glycolysis and embryonic development. **E** Targeted metabolomic profiling of the livers in geese embryos from E22, E25, and E28 (*n* = 4). **F**, **G** Dose effects of in ovo 2-DG injection in the yolk sac on lactate and ATP levels (**F**) and relative mRNA expressions of HK2, PFK, and PKM (**G**) in the liver of goose embryo (*n* = 6). **H** Developmental changes of the goose embryos after in ovo 2-DG injection in the yolk sac (*n* = 10)

The developmental pattern and correlation between embryonic growth and hepatic glycolysis were explored during the mid–late stages of goose embryogenesis (Fig. 1A). The relative body weight (RBW) and relative liver weight (RLW) increased linearly from embryonic day 16 (E16) to the day of hatching (DOH) (Fig. 1B). Hepatic glycolysis increased during the mid–late embryonic stages, based on the linear increase in the hepatic lactate content from E16 to DOH, the quadratic increase in the ATP content and the activity of HK, which peaked at E25 (Fig. 1C). Correlation analysis revealed a strong positive correlation between embryonic development and glycolysis (Fig. 1D), suggesting that E25 might be a critical window for goose embryonic development. Next, we performed targeted energy metabolomic profiling of the liver at E22, E25, and E28 (Fig. 1E and Additional file 1: Fig. S1A), which revealed that glycolysis was enhanced, as characterized by the increases in glucose 6-phosphate (G-6-P), fructose 6-phosphate (F-6-P), 3-phospho-d-glycerate (3-PG), dihydroxyacetone phosphate (DHAP), pyruvate, and lactate; however, the TCA cycle was downregulated, as characterized by the decreases in cis-aconitate, isocitrate, α-ketoglutarate, fumarate, and malic acid (Fig. 1E).

In ovo injection of 2-DG or rotenone on E24 was used to verify the dominant role of glycolysis in the energy supply during the mid–late goose embryonic stages. The dose effect of in ovo 2-DG injection showed that glycolysis was inhibited on E28 in the groups treated with 20, 40, and 80 mg/kg 2-DG, based on the decreased hepatic lactate and ATP contents (Fig. 1F), along with the decreased mRNA expression of *HK2*, *PFK*, and *PKM* (Fig. 1G). Interestingly, inhibition of glycolysis by in ovo injection of 40 or 80 mg/kg 2-DG impaired embryonic development on E28, as characterized by the lower RBW and RLW (Fig. 1H). Although there were significant decreases in the activity of mitochondrial electron transport chain complex I (Additional file 1: Fig. S1B), ATP levels (Additional file 1: Fig. S1C), and the mRNA expression of complexes I (*NDUFA5*), III (*LOC106045434*), and IV (*LOC106044242*) (Additional file 1: Fig. S1D), no adverse effects were observed in the growth (Additional file 1: Fig. S1E) and hepatic lactate levels (Additional file 1: Fig. S1C) of embryos subjected to the inhibition of mitochondrial electron transport chain function by in ovo injection of 8, 16, 32, or 64 mg/kg rotenone. In summary, glycolysis acted as the dominant mode of energy supply for the goose embryo during the mid–late developmental stages.

### Notch signaling regulates a switch to glycolysis during the mid–late embryonic stages

**Fig. 2:**
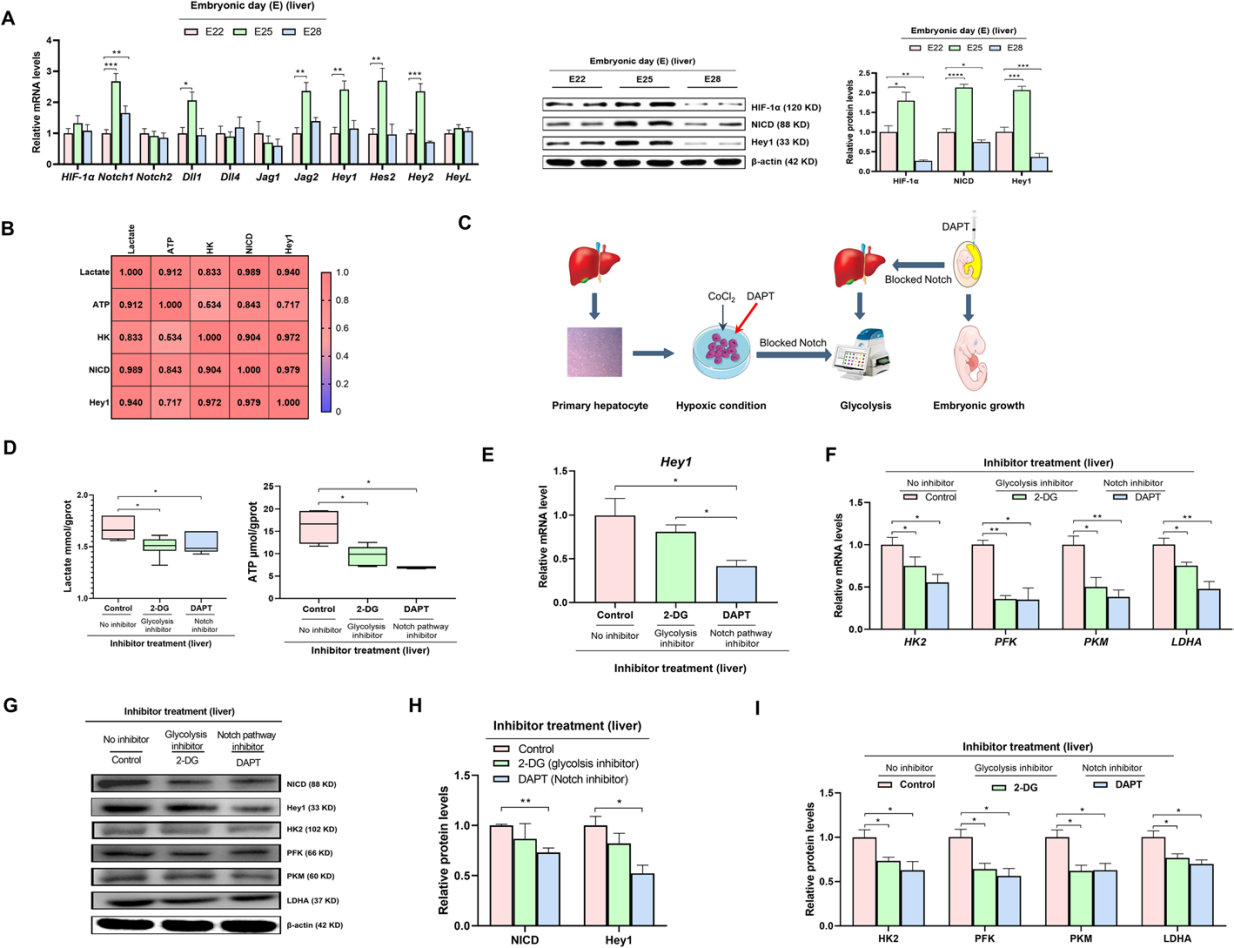
Notch signaling induced the glycolytic switch as the dominant energy supply mode during the mid–late embryonic stages. **A** Developmental changes of Notch signaling in the liver of geese embryos from E22, E25, and E28 (*n* = 6). **B** Correlation between hepatic glycolysis and Notch signaling. **C** Schematic depiction of Notch signaling regulating glycolysis in vivo and in vitro. **D** Effect of in ovo injection with 20 mg/kg 2-DG or 10 nM DAPT on lactate and ATP content in the liver (*n* = 6). **E**, **F** Effect of in ovo injection with 20 mg/kg 2-DG or 10 nM DAPT on mRNA expression of *Hey1* (**E**), *HK2*, *PFK*, *PKM*, and *LDHA* (**F**) in the liver (*n* = 6). **G**–**I** Protein abundances of Hey1 and NICD (**G**, **H**) as well as HK2, PFK, PKM, and LDHA (**G**, **I**) in the liver of the embryos in ovo injected with 20 mg/kg 2-DG or 10 nM DAPT in the yolk sac (*n* = 6)

During embryogenesis, Notch signaling pathway activity was enhanced in a quadratic manner in the embryonic liver from E22 to E28. We observed the greatest mRNA expression (*Notch1*, *Dll1*, *Jag2*, *Hey1*, *Hes2*, and *Hey2*) and protein abundance [hypoxia-inducible factor-1α (HIF-1α), Notch intracellular domain (NICD), and Hey1] on E25 (Fig. 2A). Notch signaling and glycolysis were strongly positively correlated (Fig. 2B), suggesting that Notch signaling could play an important role in the regulation of glycolysis during the mid–late embryonic stages. Based on the occurrence of a critical switch to Notch signaling during E22–E28, we performed in ovo injection of 10 nM DAPT (an inhibitor of γ-secretase) [20] on E24 (Fig. 2C) and showed inhibition of Notch signaling, as characterized by the decreased hepatic mRNA expression of *Hey1* and protein abundances of NICD and Hey1 (Fig. 2E, G, and H). Notably, and consistent with the results of in ovo 2-DG injection, in ovo DAPT injection on E24 decreased glycolysis levels on E28, as characterized by decreased hepatic lactate and ATP contents (Fig. 2D) and the decreased mRNA expression and protein abundances of HK2, PFK, PKM, and LDHA (Fig. 2F, G, and I). Impaired embryonic development but unaffected organ and intestine development was observed based on a decrease in RBW and RLW following 2-DG or DAPT injection (Additional file 1: Fig. S2A, B). No effect was found on mitochondrial function, based on the activity of mitochondrial electron transport chain complex I and the mRNA expression of complexes I (*NDUFA5*), III (*LOC106045434*), and IV (*LOC106044242*) (Additional file 1: Fig. S2C, D). However, inhibition of glycolysis by treatment with 2-DG injection did not affect Notch signaling in the embryonic liver (Fig. 2E, G, and H), suggesting that hepatic glycolytic ability could be switched by Notch signaling.

### Blocking Notch signaling suppresses glycolysis and PI3K/Akt signaling in primary hepatocytes

**Fig. 3:**
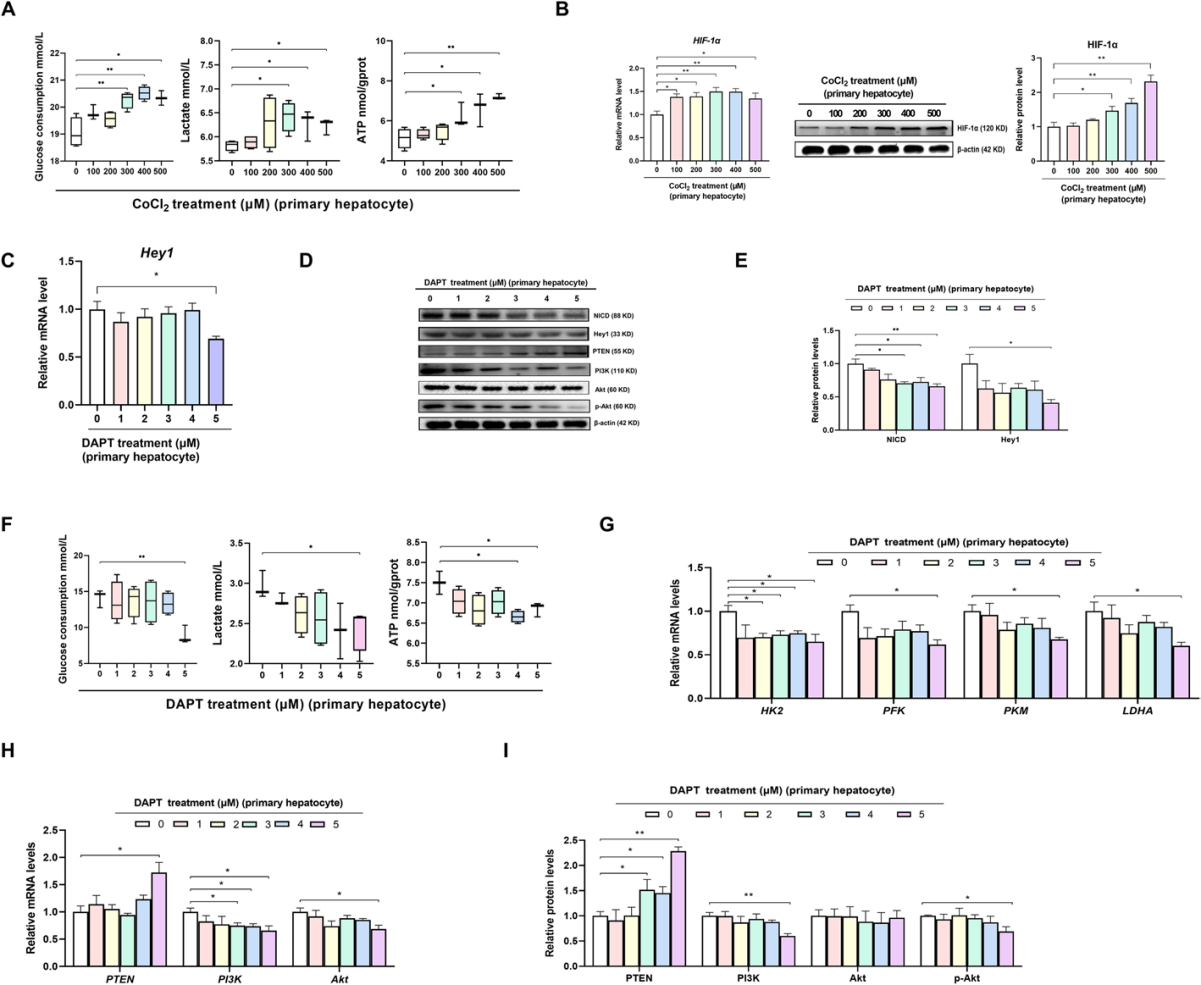
Blocking of Notch signaling reduces the hypoxic glycolysis level and the PI3K/Akt signaling in primary hepatocytes. **A**, **B** Dose effect of CoCl_2_ on glucose consumption, lactate production, and ATP levels (**A**), and the relative mRNA expression and protein abundance of HIF-1α (**B**) in goose embryonic primary hepatocytes (*n* = 6). **C**–**I** Dose effect of DAPT on Notch signaling (**C**–**F**), glucose consumption, lactate production, ATP levels (**F**), relative mRNA expressions of HK2, PFK, PKM, and LDHA (**G**), and PI3K/Akt signaling (**D**, **H**, and **I**) in goose embryonic primary hepatocytes (*n* = 6)

Primary hepatocytes were isolated from the liver tissue of geese embryos on E24 according to the procedure reported by Osman et al. [16]. Hepatocytes were identified (Additional file 1: Fig. S3A), and cell morphology (Additional file 1: Fig. S3B) and viability (Additional file 1: Fig. S3C) were evaluated. CoCl_2_ was used to simulate a physiological hypoxic incubation environment for the avian embryo. The dose effect of CoCl_2_ (300, 400, or 500 µM) in primary hepatocytes demonstrated that the HIF-1α mRNA and protein abundance (Fig. 3B), glucose consumption, lactate production, and ATP content (Fig. 3A) were increased. However, cell conjunction (Additional file 1: Fig. S4A), cell proliferation efficiency (Additional file 1: Fig. S4B), and cell viability (Additional file 1: Fig. S4C) were decreased in cells incubated with 400 or 500 µM CoCl_2_. Therefore, 300 µM CoCl_2_ (Additional file 1: Fig. S4D) was selected to induce a hypoxic culture condition for primary hepatocytes in our future study.

Under hypoxic conditions, cell conjunction (Additional file 1: Fig. S4E), cell proliferation efficiency (Additional file 1: Fig. S4F), and cell viability (Additional file 1: Fig. S4G) were decreased in cells incubated with 5 µM DAPT. The dose effect of DAPT in primary hepatocytes showed that Notch signaling was inhibited by incubation with 5 µM DAPT, as characterized by the decreased *Hey1* mRNA expression (Fig. 3C) and NICD and Hey1 protein abundances (Fig. 3D, E), along with reduced glucose consumption, lactate production, ATP content (Fig. 3F), and mRNA expression of *HK2*, *PFK*, *PKM*, and *LDHA* (Fig. 3G). No DAPT effect was found on the mRNA expression of NUDFA5, LOC106045434, or LOC1060044242 in hepatocytes (Additional file 1: Fig. S4H). These findings confirmed that glycolysis could be mediated by Notch signaling in vitro. Intriguingly, the blockade of Notch signaling also inhibited PI3K/Akt signaling, as evidenced by the decreases in the mRNA expression of *PTEN*, *PI3K*, and *Akt* and the protein abundances of PTEN, PI3K, and p-Akt (Fig. 3D, H, and I), implying that Notch signaling could mediate glycolysis in a PI3K/Akt-dependent manner.

### Notch signaling regulates glycolysis in a PI3K/Akt-dependent manner in primary hepatocytes

**Fig. 4:**
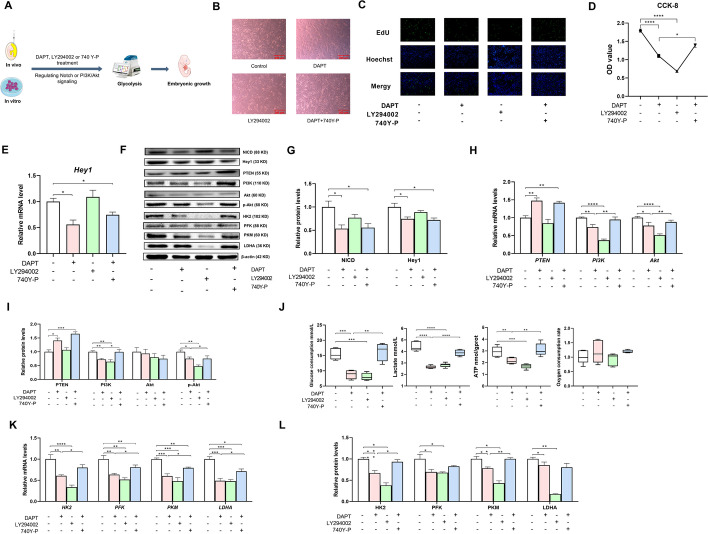
Notch signaling mediated the glycolytic switch via PI3K/Akt signaling in goose embryonic primary hepatocytes. **A** Schematic representation of the mechanism by which Notch signaling mediates the glycolytic switch to support avian embryonic growth. **B**–**D** Changes in cell morphology (**B**), cell viability (**C**), and cell proliferation (**D**) after being treated with 5 µM DAPT, 20 µM LY294002, or 20 µg/mL 740Y-P (*n* = 6). **E**–**G** Effect of 5 µM DAPT, 20 µM LY294002, or 20 µg/mL 740Y-P on Notch signaling in the goose embryonic primary hepatocytes (*n* = 6). **F**, **H**, and **I** Relative mRNA expressions of PTEN, PI3K, and Akt (**H**) and relative protein abundances of PTEN, PI3K, Akt, and p-Akt (**F**, **I**) in goose embryonic primary hepatocytes treated with 5 µM DAPT, 20 µM LY294002, or 20 µg/mL 740Y-P (*n* = 6). **F** and **J**–**L** Effect of 5 µM DAPT, 20 µM LY294002, or 20 µg/mL 740Y-P on glucose consumption, lactate production, ATP levels, oxygen consumption rate (**J**), and the relative mRNA expressions and protein abundances of HK2, PFK, PKM, and LDHA (**F**, **K** and **L**) in goose primary hepatocytes (*n* = 6)

To verify the definitive mechanism by which Notch signaling regulates glycolysis under hypoxic conditions in vitro, hepatocytes were treated with 5 µM DAPT, 20 µM LY294002 (an inhibitor available for the PI3K family) [21], or 20 µg/mL 740 Y-P (an agonist of PI3K) [22], according to previous studies (Fig. 4A). Notch signaling was inhibited by treatment of primary hepatocytes with 5 µM DAPT (Fig. 4E, F, G), whereas PI3K/Akt signaling was inhibited by treatment with 20 µM LY294002 and activated by treatment with 20 µg/mL 740 Y-P, as characterized by the increases and decreases in the mRNA and protein levels of PI3K, Akt, and p-Akt (Fig. 4F, H, I). Blocking PI3K/Akt signaling with LY294002 increased the shrinkage and exacerbated the irregular shape of hepatocytes and decreased the cell proliferation efficiency and cell viability (Fig. 4B–D). Notably, inhibition of PI3K/Akt signaling was also accompanied by a decrease in glycolysis, as evidenced by the decreases in glucose consumption, lactate production, and ATP content (Fig. 4J), along with the mRNA and protein levels of HK2, PFK, PKM, and LDHA (Fig. 4F, K, and L). In contrast, the oxygen consumption rate was unaffected, verifying that primary hepatocytes have a high dependence on glycolysis but not on oxidative phosphorylation, suggesting an important role of PI3K/Akt signaling in regulating glycolysis in primary hepatocytes. Intriguingly, activation of PI3K/AKT by 740 Y-P abrogated the DAPT-induced decrease in glycolysis, confirming that glycolysis was mediated by Notch signaling in a PI3K/Akt-dependent manner in vitro under hypoxic conditions. Moreover, the inhibitory or activating effects of DAPT, LY294002, and 740 Y-P on Notch and PI3K/Akt signaling were observed under both normal and hypoxic conditions (Additional file 1: Figs. S4I–K and S5A–C), while no effect was found on glycolysis under normal conditions (Additional file 1: Fig. S5A, D). It was suggested that Notch signaling regulates glycolysis in a PI3K/Akt-dependent manner in primary hepatocytes depending on the hypoxic condition.

### Notch signaling mediates glycolysis via PI3K/Akt to improve goose embryonic development

**Fig. 5:**
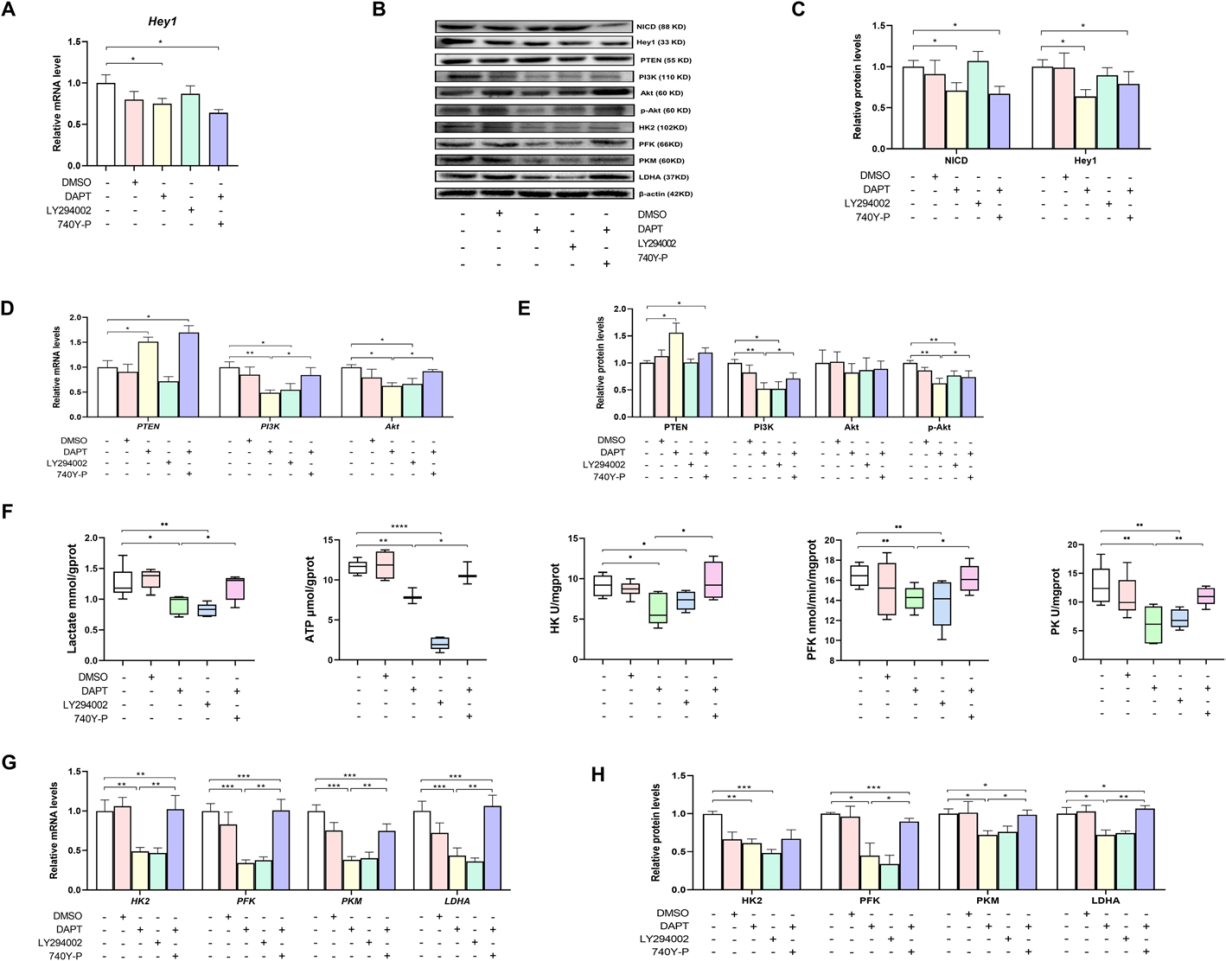
Notch signaling mediated glycolysis switch via PI3K/Akt signaling to support goose embryonic development. **A**–**E** Effect of in ovo 10 nM DAPT, 60 µM LY294002, or 50 µg/mL 740Y-P injection in the yolk sac on Notch signaling (**A**–**C**) and PI3K/Akt signaling (**B**, **D**, and **E**) in the liver of geese embryos (*n* = 6). **B** and **F**–**H** Lactate content, ATP levels, activities of HK, PFK, and PK (**F**), and the relative mRNA expressions (**G**) and protein abundances (**B**, **H**) of HK2, PFK, PKM, and LDHA in the liver of embryos in ovo injected with 10 nM DAPT, 60 µM LY294002, or 50 µg/mL 740Y-P in the yolk sac (*n* = 6)

To verify the effects of Notch signaling-mediated hepatic glycolysis on embryonic development in vivo, geese embryos were injected in ovo with 200 µL of 10 nM DAPT [20], 60 µM LY294002 [23], or 50 µg/mL 740 Y-P [24] on E24 (Fig. 4A) to inhibit Notch signaling (Fig. 5A–C) and PI3K/Akt signaling, and activate PI3K/Akt signaling (Fig. 5B, D, and E), respectively. Blockade of PI3K/Akt signaling by in ovo LY294002 injection in the yolk sac decreased hepatic glycolysis, as characterized by the decreases in the lactate and ATP contents; the activities of HK, PFK, and PKM (Fig. 5F); and the mRNA and protein levels of HK2, PFK, PKM, and LDHA (Fig. 5B, G, and H). Additionally, LY294002 treatment impaired embryonic growth on E28, as characterized by the lower RBW and RLW (Additional file 1: Fig. S6A), confirming that PI3K/Akt signaling plays an important role in glycolysis and improves goose embryonic development. Furthermore, the adverse effects on glycolysis and embryo development mediated by blockade of Notch signaling were reversed by activation of PI3K/Akt signaling (Fig. 5B, G, and H, Additional file 1: Fig. S6A, B), confirming that Notch signaling mediates glycolysis via PI3K/Akt signaling to improve goose embryonic development in vivo.

## Discussion

Cellular metabolism is switched from oxidative phosphorylation to anaerobic glycolysis under hypoxic conditions [25]. Due to the limited oxygen availability during avian embryogenesis, as observed in a naturally closed chamber [26], it was speculated that glycolysis could be the main mode of energy supply supporting the formation and growth of the avian embryo. However, the role and underlying mechanism of hepatic glycolysis in avian embryonic development remains unclear. We found that hepatic glycolysis was enhanced during E22–E28 and that inhibition of glycolysis on E24 was followed by a blockade of embryonic growth at later incubation periods. This might be because the induction of HIF-1α, subjected to the aggravated natural hypoxic environment during the incubation process, could be recruited to the Notch-responsive promoter to activate Notch signaling by the interaction between HIF-1 and NICD [27], ultimately leading to the elevated hepatic glycolysis level. However, no differences in hepatic pyruvate levels between E22 and E28 were observed, which might be due to the dynamic balance between glycolytic pyruvate production and TCA cycle pyruvate consumption [28]. The previous study showed that glycolysis supported embryonic muscle growth in *Drosophila* and zebrafish by promoting myogenic cell fusion [29]; however, a transient reduction in glycolysis reduced pathological angiogenesis [30]. In addition, Notch signaling regulates the expression of glycolysis-related genes in an environment-dependent manner in zebrafish embryonic development [31]. Therefore, no conclusive or reliable evidence exists for the role of glycolysis in avian embryonic development. Our results of in ovo 2-DG injection confirmed that the inhibition of glycolysis reduced embryonic growth in an avian embryo model, whereas the inhibition of mitochondrial electron transport chain complex I failed to impair embryonic development. Therefore, a new perspective of glycolysis in energy supply was proposed in our study: hypoxic glycolysis could act as a main energy supply mode to maintain normal development during the mid–late avian embryonic stages. Intriguingly, the inhibition of Notch signaling by in ovo DAPT injection impaired glycolytic ability and embryonic development, whereas the inhibition of glycolysis by in ovo 2-DG injection did not display significant feedback effects on Notch signaling. Our results are the first to demonstrate that there is a Notch signaling-mediated switch to glycolysis, which serves as the main energy supply mode during the mid–late embryonic stages in geese.

Previous studies reported that a switch to glycolysis can be controlled by Notch signaling via several different mechanisms [31–34]. In our study, Notch-mediated inhibition of glycolysis was accompanied by the blockade of PI3K/Akt signaling in primary hepatocytes, consistent with the finding indicating that hyperactivated Notch signaling enhanced glycolysis via activation of the PI3K/Akt signaling pathway in cancer cells [32]. In addition, PTEN is a well-known cell cycle repressor that can be negatively regulated by canonical Notch signaling [35]. As reported previously, Notch signaling has been reported to activate the PI3K/Akt pathway by inhibiting transcription of PTEN [36, 37], which was consistent with the increased PTEN mRNA and protein expression following the inhibition of Notch signaling by DAPT in the present study. Notably, the decrease in glycolysis mediated by inhibition of Notch signaling was reversed by activation of PI3K/Akt signaling. These in vitro data suggested that the switch to glycolysis was controlled by Notch signaling in a PI3K/Akt-dependent manner in embryonic primary hepatocytes. The synergism between Notch and PI3K/Akt signaling also supported the idea that Notch signaling is necessary for the regulation of glycolysis under hypoxic conditions [38] and for the maintenance of the undifferentiated cell state [27].

In our study, geese embryos were selected as an ideal avian model to verify the effect and mechanism of the Notch signaling-induced glycolytic switch in vitro. Notch signaling has been reported to be required for the development of epidermal [39] and ventricular chambers [40] during embryogenesis in mammals [41]; however, the mechanism by which Notch signaling regulates the development of avian embryos remains unclear. In our study, the reduced hepatic glycolysis resulting from the inhibition of either Notch or PI3K/Akt signaling in vivo was accompanied by impaired embryonic development, which was reversed by the activation of PI3K/Akt signaling. This finding suggests that Notch signaling induces a switch to glycolysis in the liver via PI3K/Akt signaling, which is beneficial for the embryonic development of avian species. These in vivo data provided a new perspective on the role of glycolysis in supplying energy for embryonic development; namely, a switch to hypoxic glycolysis is controlled by Notch signaling in a PI3K/Akt-dependent manner to supply energy for the mid–late embryonic stages, as demonstrated using the goose embryo model. Our findings also provide a new way to improve avian embryonic development via the regulation of either maternal dietary nutrient supplementation or in ovo feeding of exogenous nutrients. On the one hand, avian embryogenesis is important for the economic development of poultry breeding and for providing humans with high-quality animal proteins such as meat and eggs. On the other hand, avian embryos in a naturally hypoxic environment might be ideal models in developmental biology to investigate immunology, genetics, virology, and cancer under hypoxic conditions. In conclusion, our study is the first to demonstrate the role of Notch signaling in inducing a switch to glycolysis during embryonic development. Our findings present new insight into the energy supply patterns in embryogenesis under hypoxic conditions, and open up the possibility of improving avian embryonic development through exogenously mediated Notch signaling to benefit the poultry industry.

## Supplementary Information

**Additional file 1: Figure S1.** Inhibition of themitochondrial electron transport chain does not affect goose embryonicdevelopment. Metabolites besides those associated with glycolysis and theTCA cycle in the liver of goose embryos from E22, E25 and E28.Dose effectof rotenone on the activity of electron transport chain complex I, hepaticlactate and ATP levels, and the relative mRNA expressions of electrontransport chain complexes I, III, and IVin the liver of the goose embryo. Dose effect of rotenone on developmentalchanges of goose embryos. **Figure S2.** In ovoinjected with 20 mg/kg 2-DG or 10 nM DAPT impairedgoose embryonic development. Effect of in ovoinjected with 20 mg/kg 2-DG or 10 nM DAPT ondevelopmental changes of goose embryoand embryonic organs and intestines, and the activity of ETC complex I, andthe relative mRNA expressions of NUDFA5, LOC106045434, and LOC1060044242. **Figure S3.** Identification, cell morphology, and cell viability of gooseembryonic primary hepatocytes.Identification of goose embryonic primaryhepatocytes by immunofluorescence of cytokinin-8and cytokinin-18.CK18 and CK18, DAPI, scale bar: 50 μm. Morphology of gooseembryonic primary hepatocytes from 0 to 72 h after isolation.Changes incell viability within one week of goose embryo primary hepatocytes. **Figure S4.** Dose effect of CoCl_2_ or DAPT on goose embryonic primaryhepatocytes. Dose effect of CoCl_2_ on cell morphology, cellproliferation, and cell viability. Hepatocyte viability within 1week after being treated with 300 μM CoCl_2_. Dose effect ofDAPT on cell morphology, cell proliferation, cell viability, andthe relative mRNA expressions of NUDFA5, LOC106045434, and LOC1060044242. Effect of CoCl_2_ and DAPT onrelative protein abundances of NICD, Hey1, PTEN, PI3K, Akt, and p-Akt inhepatocytes under normal condition. **Figure S5.** Effects of DAPT, LY294002 and 740Y-Ptreatment on Notch signaling, PI3K/Akt signaling, and glycolysisin hepatocytes under normal condition. **Figure S6.** In ovoinjected with 10 nM DAPT or 60 μM LY294002impaired goose embryonic development. Effect of in ovo10 nM DAPT, 60 μMLY294002, or 50 μg/mL 740Y-P injection on developmental changes of gooseembryosand embryonic organs and intestines.

## Data Availability

The datasets generated during and/or analyzed during the current study are available from the corresponding author on reasonable request.

## Abbreviations

DHAP: Dihydroxyacetone phosphate
DOH: Day of hatching
DAPT: *N*-[*N*-(3,5-Difluorophenacetyl)-l-alanyl]-*S*-phenyl glycine t-butyl ester
EdU: 5-Ethynyl-2′-deoxyuridine
E: Embryonic day
F-6-P: Fructose 6-phosphate
G-6-P: Glucose 6-phosphate
HIF-1α: Hypoxia-inducible factor-1α
HK: Hexokinase
NICD: Notch intracellular domain
PFK: Phosphofructokinase
PK: Pyruvate kinase
RQ: Respiratory quotient
RBW: Relative body weight
RLW: Relative liver weight
2-DG: 2-Deoxy-d-glucose
3-PG: 3-Phospho-d-glycerate

## References

[1] Hou S, Liu L (2021). Development status, future development trends and suggestions of the waterfowl industry in 2020. Chin J Anim Sci.

[2] Yadgary L, Cahaner A, Kedar O, Uni Z (2010). Yolk sac nutrient composition and fat uptake in late-term embryos in eggs from young and old broiler breeder hens. Poult Sci.

[3] Christensen VL, Grimes JL, Donaldson WE, Lerner S (2000). Correlation of body weight with hatchling blood glucose concentration and its relationship to embryonic survival. Poult Sci.

[4] van der Wagt I, de Jong IC, Mitchell MA, Molenaar R, van den Brand H (2020). A review on yolk sac utilization in poultry. Poult Sci.

[5] Foye OT, Uni Z, Ferket PR (2006). Effect of in ovo feeding egg white protein, beta-hydroxy-beta-methylbutyrate, and carbohydrates on glycogen status and neonatal growth of turkeys. Poult Sci.

[6] Uni Z, Ferket PR, Tako E, Kedar O (2005). In ovo feeding improves energy status of late-term chicken embryos. Poult Sci.

[7] De Oliveira JE, Uni Z, Ferket PR (2008). Important metabolic pathways in poultry embryos prior to hatch. World’s Poult Sci J.

[8] Choi CS, Fillmore JJ, Kim JK, Liu Z, Kim S, Collier EF (2007). Overexpression of uncoupling protein 3 in skeletal muscle protects against fat-induced insulin resistance. J Clin Invest.

[9] Moran ETJ (2007). Nutrition of the developing embryo and hatchling. Poult Sci.

[10] Li M, Cheng R, Liang J, Yan H, Zhang H, Yang L (2013). Mutations in pofut1, encoding protein o-fucosyltransferase 1, cause generalized dowling-degos disease. Am J Hum Genet.

[11] Andersson ER, Sandberg R, Lendahl U (2011). Notch signaling: simplicity in design, versatility in function. Development.

[12] Bigas A, Robert-Moreno A, Espinosa L (2010). The Notch pathway in the developing hematopoietic system. Int J Dev Biol.

[13] Bahrampour S, Thor S. The five faces of notch signalling during *Drosophila melanogaster* embryonic CNS development No. 1218. 2020. p. 39–58. 10.1007/978-3-030-34436-8_3 32060870

[14] Guo B, Yan L, Lei M, Dai Z, Shi Z (2021). Wider angle egg turning during incubation enhances yolk utilization and promotes goose embryo development. Animals.

[15] Feng Z, Gong H, Fu J, Xu X, Song Y, Yan X (2022). In ovo injection of chir-99021 promotes feather follicle development via modulating the wnt signaling pathway and transcriptome in goose embryos (*Anser cygnoides*). Front Physiol.

[16] Osman RH, Shao D, Liu L, Xia L, Sun X, Zheng Y (2016). Expression of mitochondria-related genes is elevated in overfeeding-induced goose fatty liver. Comp Biochem Physiol B Biochem Mol Biol.

[17] Ghaedi M, Soleimani M, Shabani I, Duan Y, Lotfi AS (2012). Hepatic differentiation from human mesenchymal stem cells on a novel nanofiber scaffold. Cell Mol Biol Lett.

[18] Liu Y, Wang X, Zhen Z, Yu Y, Qiu Y, Xiang W (2019). GRP78 regulates milk biosynthesis and the proliferation of bovinemammaryepithelial cells through the mTOR signaling pathway. Cell Mol Biol Lett.

[19] Muñoz Sánchez J, Chánez Cárdenas ME (2018). The use of cobalt chloride as a chemical hypoxia model. J Appl Toxicol.

[20] Zuo Q, Zhang C, Jin K, Jing J, Sun C, Ahmed MF (2018). NICD-mediated notch transduction regulates the different fate of chicken primordial germ cells and spermatogonial stem cells. Cell Biosci.

[21] Song Q, Han CC, Xiong XP, He F, Gan W, Wei SH (2016). PI3K-Akt-mTOR signal inhibition affects expression of genes related to endoplasmic reticulum stress. Genet Mol Res.

[22] Liang Y, Pan Q, Wang R, Ye Z, Li Z, Zeng L (2019). Microvesicles derived from tgf-β1 stimulated hepatic stellate cells aggravate hepatocellular injury. Stem Cells Dev.

[23] Hu Y, Poopalasundaram S, Graham A, Bouloux P (2013). GnRH neuronal migration and olfactory bulb neurite outgrowth are dependent on FGF receptor 1 signaling, specifically via the PI3K p110α isoform in chick embryo. Endocrinology.

[24] Sun C, Zhang Z, He P, Zhou Y, Xie X (2017). Involvement of PI3K/Akt pathway in the inhibition of hepatocarcinoma cell invasion and metastasis induced by sash1 through downregulating shh-gli1 signaling. Int J Biochem Cell Biol.

[25] Denko NC, Hypoxia (2008). HIF1 and glucose metabolism in the solid tumour. Nat Rev Cancer.

[26] Uni Z, Ferket RP (2004). Methods for early nutrition and their potential. World's Poult Sci J.

[27] Gustafsson MV, Zheng X, Pereira T, Gradin K, Jin S, Lundkvist J (2005). Hypoxia requires Notch signaling to maintain the undifferentiated cell state. Dev Cell.

[28] Shannon CE, Ragavan M, Palavicini JP, Fourcaudot M, Bakewell TM, Valdez IA, Ayala I, Jin ES, Madesh M, Han X, Merritt ME, Norton L (2021). Insulin resistance is mechanistically linked to hepatic mitochondrial remodeling in non-alcoholic fatty liver disease. Mol Metab.

[29] Tixier V, Bataillé L, Etard C, Jagla T, Weger M, DaPonte JP (2013). Glycolysis supports embryonic muscle growth by promoting myoblast fusion. Proc Natl Acad Sci USA.

[30] Schoors S, De Bock K, Cantelmo AR, Georgiadou M, Ghesquiere B, Cauwenberghs S (2014). Partial and transient reduction of glycolysis by PFKFB3 blockade reduces pathological angiogenesis. Cell Metab.

[31] Kuwabara S, Yamaki M, Yu H, Itoh M (2018). Notch signaling regulates the expression of glycolysis-related genes in a context-dependent manner during embryonic development. Biochem Biophys Res Commun.

[32] Landor SK, Mutvei AP, Mamaeva V, Jin S, Busk M, Borra R (2011). Hypo- and hyperactivated Notch signaling induce a glycolytic switch through distinct mechanisms. Proc Natl Acad Sci USA.

[33] Moriyama H, Moriyama M, Ozawa T, Tsuruta D, Iguchi T, Tamada S (2018). Notch signaling enhances stemness by regulating metabolic pathways through modifying p53, NF-κB, and HIF-1α. Stem Cells Dev.

[34] Slaninova V, Krafcikova M, Perez-Gomez R, Steffal P, Trantirek L, Bray SJ (2016). Notch stimulates growth by direct regulation of genes involved in the control of glycolysis and the tricarboxylic acid cycle. Open Biol.

[35] Marathe S, Liu S, Brai E, Kaczarowski M, Alberi L (2015). Notch signaling in response to excitotoxicity induces neurodegeneration via erroneous cell cycle reentry. Cell Death Differ.

[36] Zhang X, Yang Y, Feng Z (2018). Suppression of microRNA-495 alleviates high-glucose-induced retinal ganglion cell apoptosis by regulating Notch/PTEN/Akt signaling. Biomed Pharmacother.

[37] Vaish V, Sanyal SN (2012). Role of Sulindac and Celecoxib in the regulation of angiogenesis during the early neoplasm of colon: exploring PI3-K/PTEN/Akt pathway to the canonical Wnt/beta-catenin signaling. Biomed Pharmacother.

[38] Moriyama H, Moriyama M, Isshi H, Ishihara S, Okura H, Ichinose A (2014). Role of Notch signaling in the maintenance of human mesenchymal stem cells under hypoxic conditions. Stem Cells Dev.

[39] Moriyama M, Durham AD, Moriyama H, Hasegawa K, Nishikawa S, Radtke F (2008). Multiple roles of Notch signaling in the regulation of epidermal development. Dev Cell..

[40] Grego-Bessa J, Luna-Zurita L, Del Monte G, Bolós V, Melgar P, Arandilla A (2007). Notch signaling is essential for ventricular chamber development. Dev Cell.

[41] Li S, Shi Y, Dang Y, Luo L, Hu B, Wang S (2021). Notch signaling pathway is required for bovine early embryonic development. Biol Reprod.

