# Supplementary material for: Notch mediates the glycolytic switch via PI3K/Akt signaling to support embryonic development
Source: Cell Mol Biol Lett. 2023 Jun 26;28:50. doi: 10.1186/s11658-023-00459-4 (PMC10294521; doi:10.1186/s11658-023-00459-4)
Supplement: Supplementary file 1 — Additional file 1: Figure S1. Inhibition of themitochondrial electron transport chain does not affect goose embryonicdevelopment. Metabolites besides those associated with glycolysis and theTCA cycle in the liver of goose embryos from E22, E25 and E28.Dose effectof rotenone on the activity of electron transport chain complex I, hepaticlactate and ATP levels, and the relative mRNA expressions of electrontransport chain complexes I, III, and IVin the liver of the goose embryo. Dose effect of rotenone on developmentalchanges of goose embryos. Figure S2. In ovoinjected with 20 mg/kg 2-DG or 10 nM DAPT impairedgoose embryonic development. Effect of in ovoinjected with 20 mg/kg 2-DG or 10 nM DAPT ondevelopmental changes of goose embryoand embryonic organs and intestines, and the activity of ETC complex I, andthe relative mRNA expressions of NUDFA5, LOC106045434, and LOC1060044242. Figure S3. Identification, cell morphology, and cell viability of gooseembryonic primary hepatocytes.Identification of goose embryonic primaryhepatocytes by immunofluorescence of cytokinin-8and cytokinin-18.CK18 and CK18, DAPI, scale bar: 50 μm. Morphology of gooseembryonic primary hepatocytes from 0 to 72 h after isolation.Changes incell viability within one week of goose embryo primary hepatocytes. Figure S4. Dose effect of CoCl2 or DAPT on goose embryonic primaryhepatocytes. Dose effect of CoCl2 on cell morphology, cellproliferation, and cell viability. Hepatocyte viability within 1week after being treated with 300 μM CoCl2. Dose effect ofDAPT on cell morphology, cell proliferation, cell viability, andthe relative mRNA expressions of NUDFA5, LOC106045434, and LOC1060044242. Effect of CoCl2 and DAPT onrelative protein abundances of NICD, Hey1, PTEN, PI3K, Akt, and p-Akt inhepatocytes under normal condition. Figure S5. Effects of DAPT, LY294002 and 740Y-Ptreatment on Notch signaling, PI3K/Akt signaling, and glycolysisin hepatocytes under normal condition. Figure S6. In ovoinjected with 10 nM DA [file 11658_2023_459_MOESM1_ESM.pdf]

Additional file 1

Notch mediates the glycolytic switch via PI3K/Akt signaling to support the embryonic development

Heng Wang, Wenqi Liang, Xuyang Wang, Yuchun Zhan, Wence Wang, Lin Yang, Yongwen Zhu

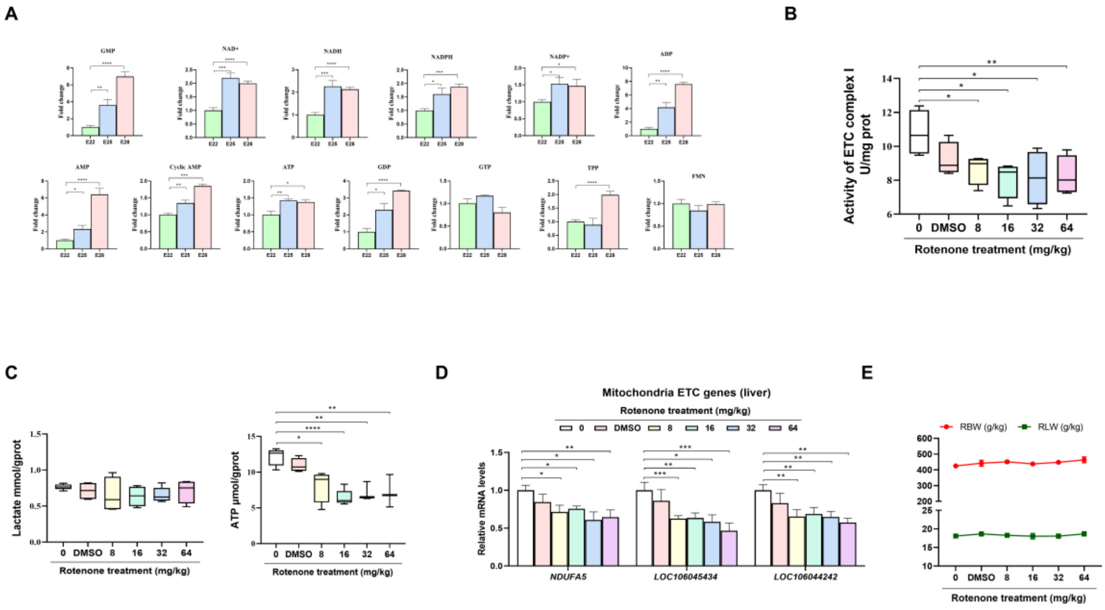

Fig S1. Inhibition of the mitochondrial electron transport chain does not affect goose embryonic development. (A) Metabolites besides those associated with glycolysis and the TCA cycle in the liver of goose embryos from E22, E25 and E28 (n = 4). (B-E) Dose effect of rotenone on the activity of electron transport chain complex I (B), hepatic lactate and ATP levels (C), and the relative mRNA expressions of electron transport chain complexes I, III, and IV (D) in the liver of the goose embryo (n = 6). (E) Dose effect of rotenone on developmental changes of goose embryos (n = 10).

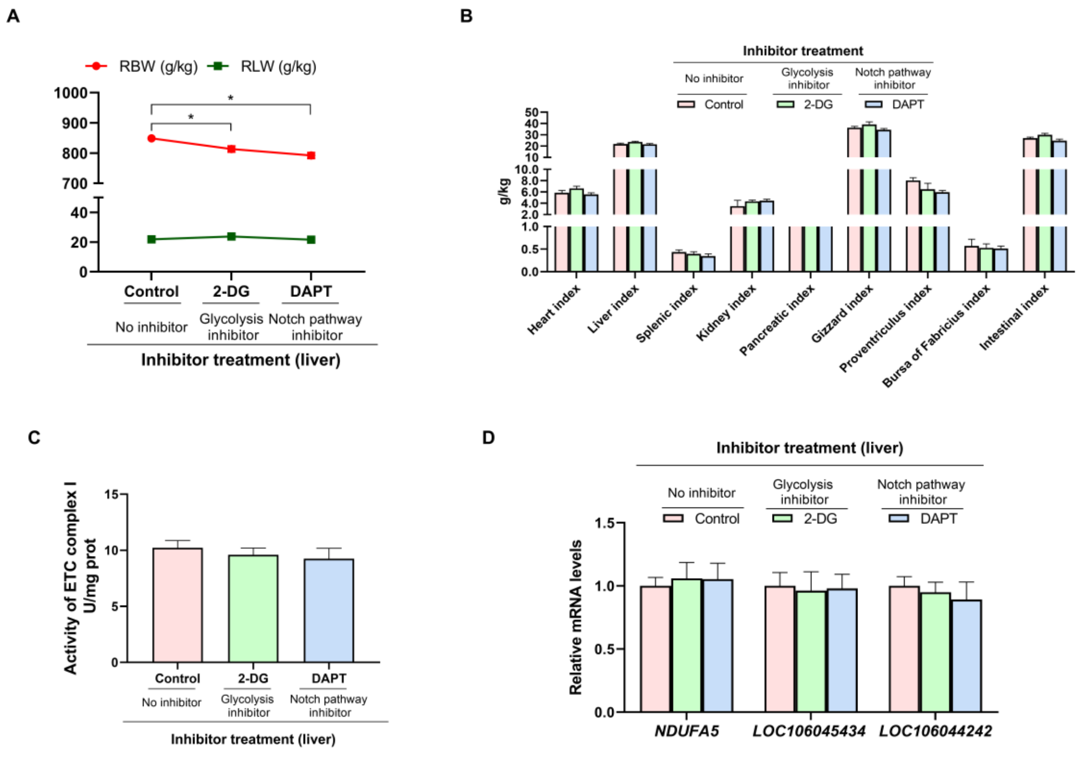

Fig S2. *In ovo* injected with 20 mg/kg 2-DG or 10 nM DAPT impaired goose embryonic development. (A, B, C, and D) Effect of *in ovo* injected with 20 mg/kg 2-DG or 10 nM DAPT on developmental changes of goose embryo (A) and embryonic organs and intestines (B) (n = 10), and the activity of ETC complex I (C), and the relative mRNA expressions of NUDFA5, LOC106045434, and LOC1060044242 (D) (n = 6).

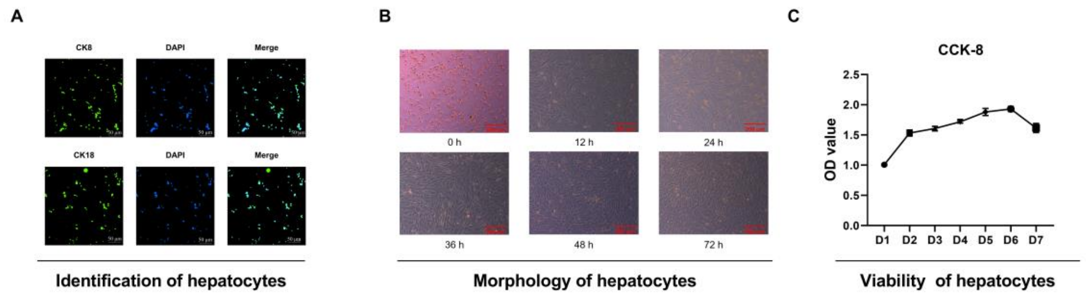

Fig S3. Identification, cell morphology, and cell viability of goose embryonic primary hepatocytes. (A) Identification of goose embryonic primary hepatocytes by immunofluorescence of cytokinin-8 (CK8) and cytokinin-18 (CK18). CK18 and CK18 (green), DAPI (blue), scale bar: 50 μm. (B) Morphology of goose embryonic primary hepatocytes from 0 to 72 h after isolation. (C) Changes in cell viability within one week of goose embryo primary hepatocytes (n = 6).

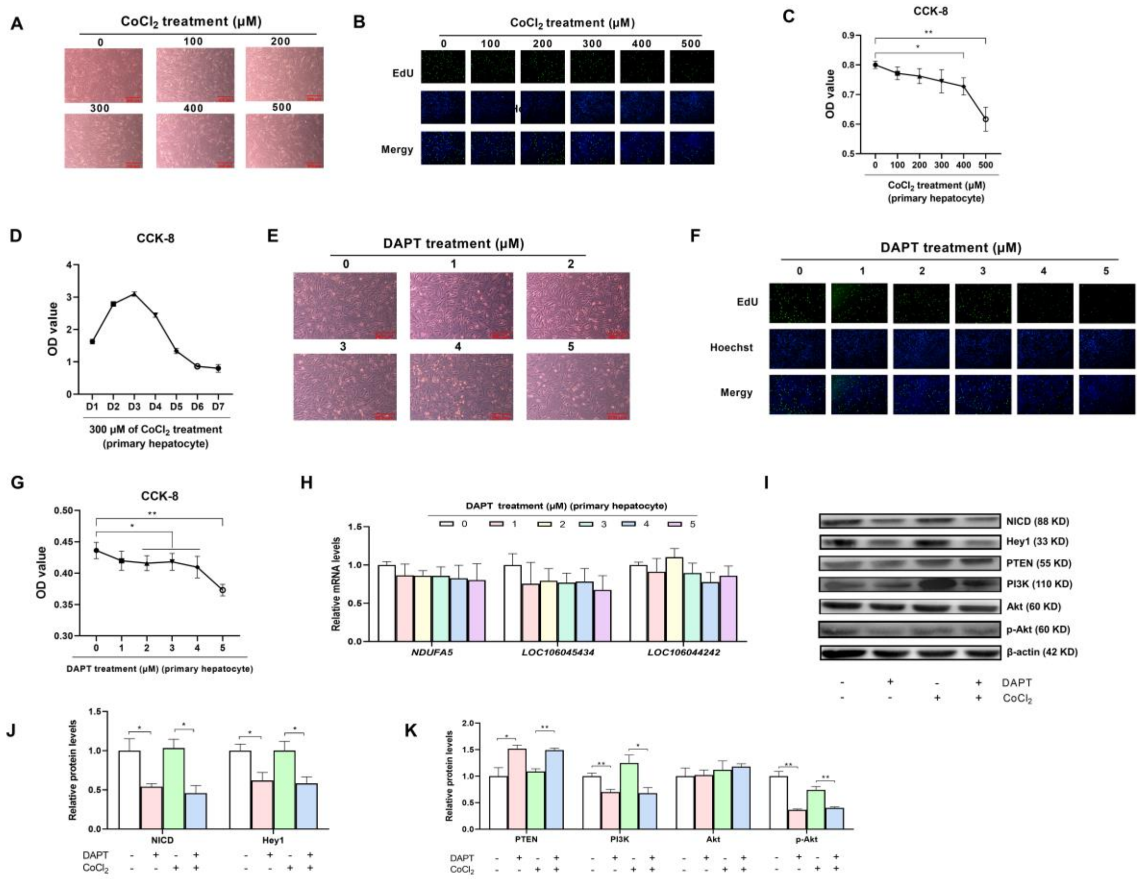

Fig S4. Dose effect of $CoCl_2$ or DAPT on goose embryonic primary hepatocytes. (A, B, and C) Dose effect of $CoCl_2$ on cell morphology (A), cell proliferation (B), and cell viability (C). (D) Hepatocyte viability within one week after being treated with 300 μM $CoCl_2$. (E-H) Dose effect of DAPT on cell morphology (E), cell proliferation (F), cell viability (G), and the relative mRNA expressions of NUDFA5, LOC106045434, and LOC1060044242 (H) (n = 6). (I-K) Effect of $CoCl_2$ and DAPT on relative protein abundances of NICD, Hey1, PTEN, PI3K, Akt, and p-Akt in hepatocytes under normal condition (n = 6).

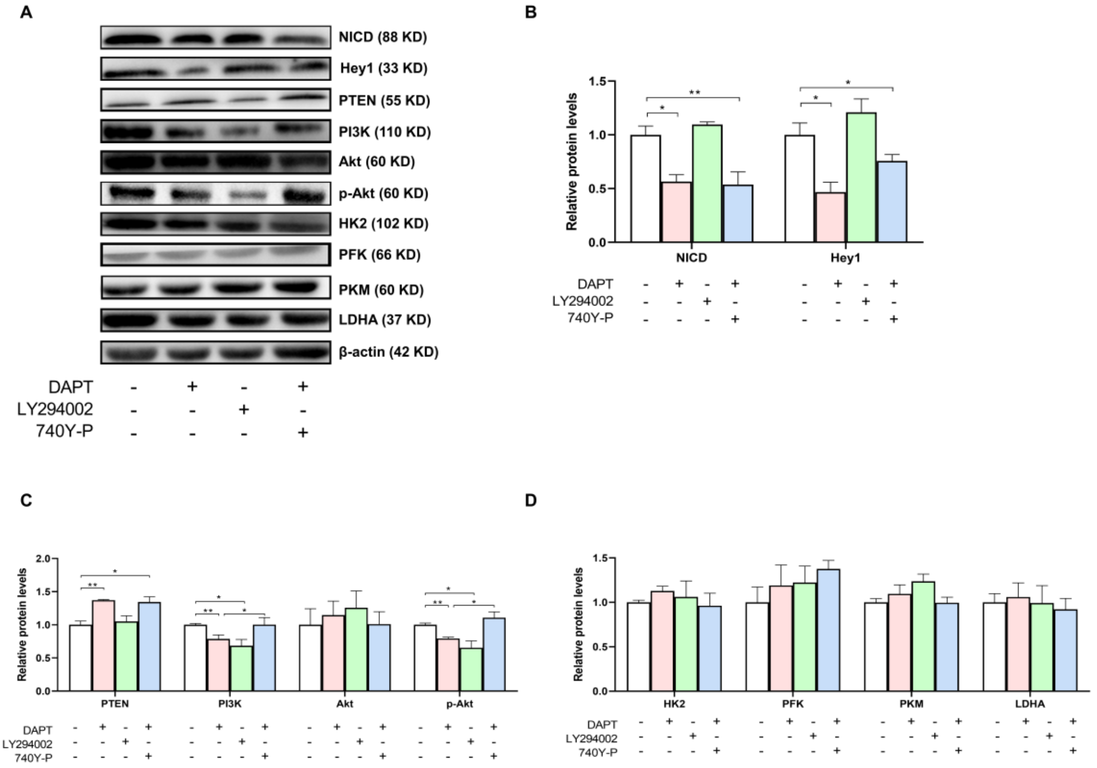

Fig S5. Effects of DAPT, LY294002 and 740Y-P treatment on Notch signaling (A, B), PI3K/Akt signaling (A, C), and glycolysis (A, D) in hepatocytes under normal condition (n = 6).

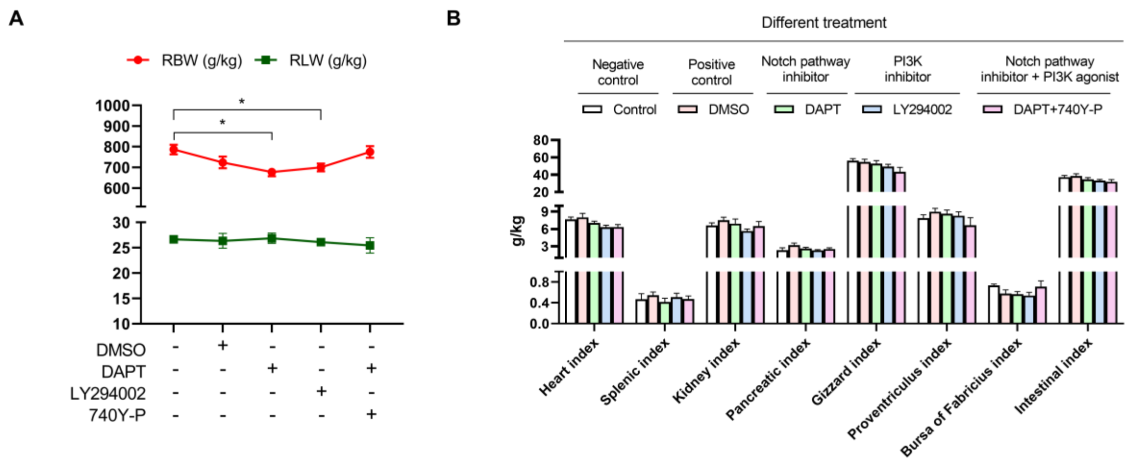

Fig S6. *In ovo* injected with 10 nM DAPT or 60 μM LY294002 impaired goose embryonic development. (A and B) Effect of *in ovo* 10 nM DAPT, 60 μM LY294002, or 50 μg/mL 740Y-P injection on developmental changes of goose embryos (A) and embryonic organs and intestines (B) (n = 10).
